# AURKB promotes colorectal cancer progression by triggering the phosphorylation of histone H3 at serine 10 to activate CCNE1 expression

**DOI:** 10.18632/aging.205801

**Published:** 2024-05-06

**Authors:** Ling Li, Ke Xie, Honghu Xie, Lei Wang, Zhong Li, Qicheng Lu, Jin Feng

**Affiliations:** 1 Department of Gastrointestinal Surgery, The First People’s Hospital of Changzhou, The Third Affiliated Hospital of Soochow University, Changzhou, China

**Keywords:** colorectal cancer, AURKB, CCNE1, cell cycle, polyploidy

## Abstract

Aurora kinase B (AURKB) initiates the phosphorylation of serine 10 on histone H3 (pH3S10), a crucial process for chromosome condensation and cytokinesis in mammalian mitosis. Nonetheless, the precise mechanisms through which AURKB regulates the cell cycle and contributes to tumorigenesis as an oncogenic factor in colorectal cancer (CRC) remain unclear. Here, we report that AURKB was highly expressed and positively correlated with Ki-67 expression in CRC. The abundant expression of AURKB promotes the growth of CRC cells and xenograft tumors in animal model. AURKB knockdown substantially suppressed CRC proliferation and triggered cell cycle arrest in G2/M phase. Interestingly, cyclin E1 (CCNE1) was discovered as a direct downstream target of AURKB and functioned synergistically with AURKB to promote CRC cell proliferation. Mechanically, AURKB activated CCNE1 expression by triggering pH3S10 in the promoter region of CCNE1. Furthermore, it was showed that the inhibitor specific for AURKB (AZD1152) can suppress CCNE1 expression in CRC cells and inhibit tumor cell growth. To conclude, this research demonstrates that AURKB accelerated the tumorigenesis of CRC through its potential to epigenetically activate CCNE1 expression, suggesting AURKB as a promising therapeutic target in CRC.

## INTRODUCTION

Colorectal cancer (CRC) ranks as the third major contributor to cancer-related fatalities across the globe [1]. The occurrence rate of CRC is on the decline due to timely detection and swift elimination of pre-cancerous lesions; however, the outlook remains unfavorable for advanced and metastatic stages of CRC [2]. Chemotherapy is considered the established therapy for individuals diagnosed with advanced CRC [3]. However, the emergence of tumor cell resistance to chemotherapy, coupled with potential systemic toxicity, is constraining the effectiveness of treatment [4]. Therefore, there is an urgent requirement for novel and focused therapeutic alternatives.

Aurora kinase B (AURKB), which constitutes the enzymatic core of the chromosomal passenger complex (CPC), plays a pivotal role in overseeing accurate chromosome segregation by governing various stages of cell division, including chromosome condensation and cohesion, attachments between kinetochores and microtubules, the spindle assembly checkpoint, and cytokinesis [5]. AURKB is positioned at the centromeres starting from prophase and lasting through the transition from metaphase to anaphase, and it plays a significant role in cell division that predominantly occurs between the G2 phase and the M phase [6, 7]. Prior research suggests that AURKB initiates the phosphorylation of histone H3 at serine 10 (pH3S10), a process linked to the stability of chromosome numbers and the compaction of chromatin during mitosis [8, 9]. Inhibiting AURKB directly hampers the process of cytokinesis and leads to severe abnormalities during mitosis [10, 11]. Increased levels of AURKB could lead to a decrease in the presence of the cell-cycle inhibitor p21 by inhibiting the activity of p53, which in turn could cause abnormal activation of cyclin-dependent kinase 1 (Cdk1) [12]. Alternatively, AURKB could potentially phosphorylate Class IIa histone deacetylases directly, triggering the activation of the AKT/mTOR signaling pathway to facilitate cell-cycle progression, ultimately promote cell proliferation [13, 14]. AURKB expression is found to be aberrantly elevated in multiple types of cancers, such as breast cancer [15], prostate cancer [16], and lung cancer [17], indicating its possible potential as an oncogene. Nevertheless, the role of AURKB in human cancer biology is largely unclarified.

The current study aims to elucidate the biological function and its functioning mechanism during CRC progression. It was hypothesized that AURKB is abundantly expressed in CRC cells and tissues, which accelerated the cell cycle progression of tumor cells through its regulation on the expression of key cycle regulators.

## MATERIALS AND METHODS

### Bioinformatics analysis

The gene expression profile dataset GSE74602 which contains the gene expression data of 30 paired normal and tumor colorectal samples was downloaded from GEO database (https://www.ncbi.nlm.nih.gov/geo/). Platforms of GEO dataset is GPL6104 (Illumina humanRef-8 v2.0 expression beadchip). The expression pattern of AURKB in Colon adenocarcinoma (COAD) and Rectum adenocarcinoma (READ) in TCGA database were obtained from GEPIA 2 website (http://gepia2.cancer-pku.cn/). Representative immunochemistry staining images of AUTKB-positive cells between CRC tissues and normal rectum or colon tissues were obtained from The Human Protein Atlas database (http://www.proteinatlas.org).

### Cell culture

CRC cell lines (HCT116, SW620, SW480, HT29, LOVO) were cultured at 37° C in a humidified incubator with 5% CO_2_.

### Cell transfection

To stably knockdown AURKB, shRNA targeting AURKB (shAURKB) and scrambled sequences (shNC) were inserted into the pLVX-IRES-mCherry vector. HCT116 and SW620 cells were infected with the viral products from the HEK293T cells. To overexpress CCNE1, human CCNE1 cDNA was cloned into the retroviral vector MSCV-IRES-HA-GFP. AURKB-silenced cells were infected with the retrovirus product.

### AZD1152 treatment

AZD1152-HQPA, the active metabolite of AZD1152, was obtained from Sigma-Aldrich (USA) and was dissolved in DMSO to incubate with the designated cells.

### RT-qPCR

Total RNA was isolated from CRC cell lines and tissues using TRIzol (Invitrogen, USA). cDNA was generated using a reverse transcription kit (Takara, China). GAPDH was employed as an internal control to determine relative mRNA expression levels through the comparative Ct method. The primer sequences used in RT-PCR are listed as follows.

AURKB forward GGAGTGCTTTGCTATGAGCTGC, reverse GAGCAGTTTGGAGATGAGGTCC;

CCND1 forward TCTACACCGACAACTCCATCCG, reverse TCTGGCATTTTGGAGAGGAAGTG;

CDK2 forward ATGGATGCCTCTGCTCTCACTG, reverse CCCGATGAGAATGGCAGAAAGC;

CDK4 forward CCATCAGCACAGTTCGTGAGGT, reverse TCAGTTCGGGATGTGGCACAGA;

CDKN1A forward AGGTGGACCTGGAGACTCTCAG, reverse TCCTCTTGGAGAAGATCAGCCG;

CCNE1 forward TGTGTCCTGGATGTTGACTGCC, reverse CTCTATGTCGCACCACTGATACC;

GAPDH forward GTCTCCTCTGACTTCAACAGCG, reverse ACCACCCTGTTGCTGTAGCCAA.

### Western blot

Protein was extracted from tissue samples or cells, and the protein concentrations were measured using a BCA assay kit (Beyotime Biotechnology, China). Equal amounts of protein were separated using SDS-PAGE and transferred to PVDF membranes (Millipore, USA). Primary antibodies used include anti-pH3S10, anti-H3, anti-AURKB, anti-CCNE1, and anti-GAPDH. Horseradish peroxidase (HRP)-conjugated secondary antibodies were used and the protein signals were visualized using ECL detection reagents (Millipore, USA).

### Cell counting kit-8 (CCK-8) and colony formation assays

Cell proliferation was assessed using a CCK-8 kit (Dojindo, Japan) to measure cell viability. Briefly, cells were seeded in 96-well plates followed by addition of CCK-8 reagent at indicated time points. The absorbance was determined using a spectrophotometer at 450 nm. To calculate the half-maximal inhibitory concentration (IC_50_) values, cells were exposed to increasing concentrations of AZD1152-HQPA for 24 h. For colony formation, cells were seeded into six-well plates. Cell colonies were observed by fixing and staining the cells using crystal violet two weeks later.

### Flow cytometry analysis

Cell cycle was analyzed using the 647 EdU Click Proliferation Kit (BD Bioscience, USA), and the apoptotic cell death was evaluated by Annexin V-FITC staining. The samples were assessed using flow cytometry to determine apoptosis (Annexin V-positive) and to analyze the two-dimensional cell cycle distribution (EdU-Alexa647 and DAPI) using a BD LSRFortessa flow cytometer from BD Bioscience. Data from flow cytometry were analyzed using FlowJo software.

### Xenograft tumor model

To create a tumor-bearing model, HCT116 cells were subcutaneously injected into the right flank of male nude mice (6-week-old, Shanghai SLAC Laboratory Animal Co., Ltd., China). The size of the tumors was assessed every three days using Vernier calipers, and the tumor volume was computed using the formula: volume = length × width^2 × 0.5. After a period of four weeks, all the experimental mice were humanely euthanized, and the tumors were removed, then preserved in formalin.

### Chromatin immunoprecipitation (ChIP) assay

ChIP assay was carried out as described previously [18]. Briefly, the cells were rinsed with 1×PBS and treated with 1% formaldehyde (Sigma-Aldrich) for crosslinking. Subsequently, they were subjected to sonication to produce DNA fragments with an average length ranging from 200 to 500 base pairs. Soluble chromatin was then incubated with an antibody against pH3S10 overnight at 4° C, and immunoprecipitation was performed with Protein A/G beads. RT-qPCR was performed using precipitated DNA as the template.

### Statistical analysis

All data are displayed as the mean ± standard deviation (SD) of samples in three-independent experiments. Student’s t-test was used to determine the statistical significance between groups and a p-value < 0.05 was considered as statistically significant.

## RESULTS

### AURKB was identified as a potent oncogene in CRC

**Figure 1:**
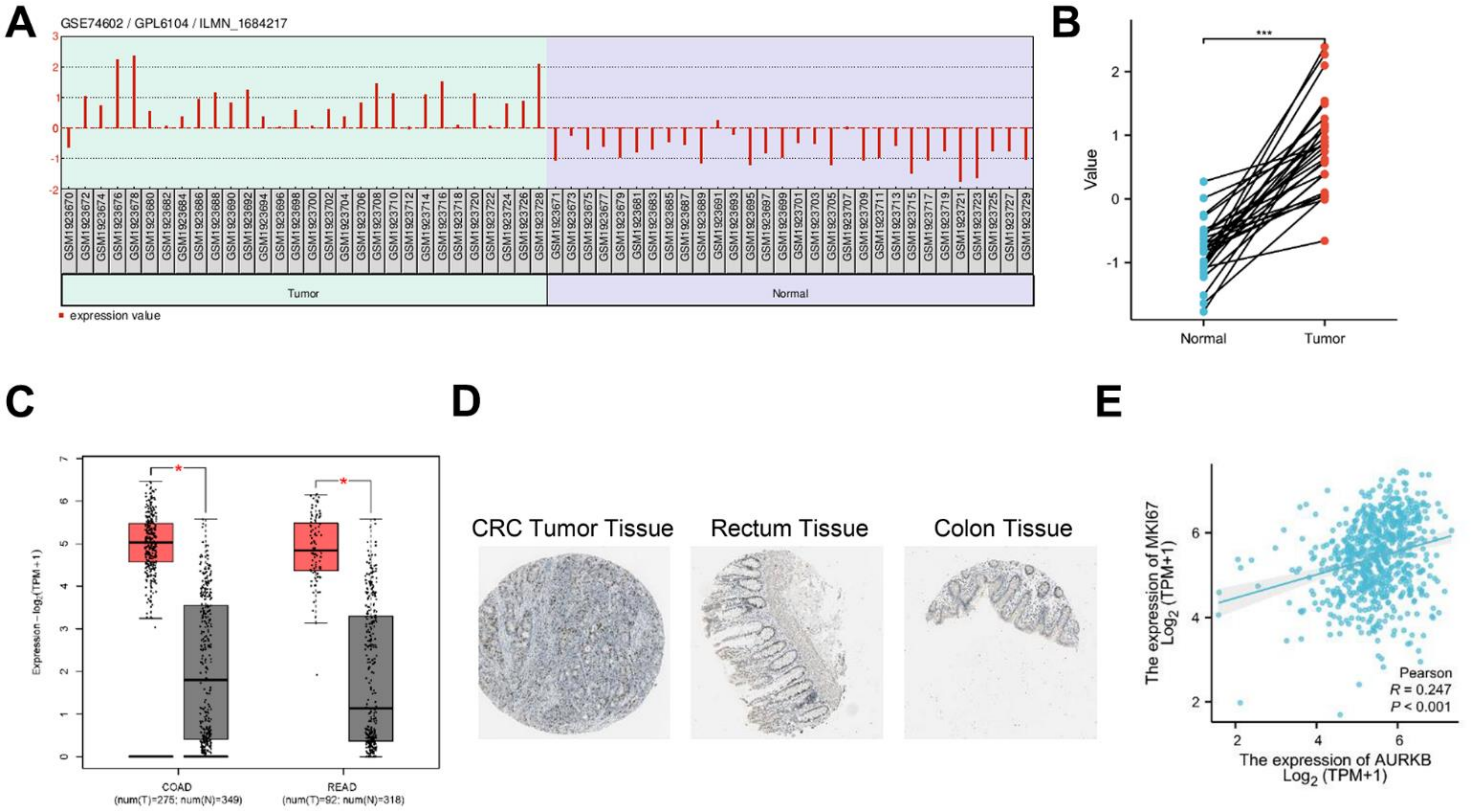
**AURKB was identified as a potent oncogene in CRC.** (**A**) Expression profile of AURKB in GEO dataset GSE74602. (**B**) Matched comparison of AURKB expression between tumor and non-tumor tissues in GSE74602 dataset. (**C**) The mRNA expression between tumor and normal tissues was compared between TCGA Colon adenocarcinoma (COAD) and Rectum adenocarcinoma (READ) databases. (**D**) Representative images of AURKB staining in CRC tumor tissues and normal colon and rectum tissues were obtained from HPA database (https://www.proteinatlas.org/). (**E**) The correlation between AURKB and Ki-67 expression in CRC tissues was obtained from TCGA database using cBioPortal website (https://www.cbioportal.org/).

To explore the role of AURKB in CRC, we first interrogated GEO dataset GSE74602 which compares the gene expression between 30 pairs of colon tumor tissue and corresponding non-cancerous tissue. As shown in Figure 1A, 1B, AURKB was significantly upregulated in tumor tissues. Similarly, TCGA database indicated that the mRNA levels of AURKB were substantially elevated in CRC tissues (Figure 1C). Immunohistochemical data from the HPA project showed higher staining and stronger intensity of AURKB antibody in CRC tissues [19] than in normal colon and rectum tissues [20, 21]. In addition, the expression of AURKB and Ki-67 was positively correlated in TCGA CRC tumor tissues (Figure 1E). These data suggested that AURKB was abundantly expressed in CRC and may function as an oncogene.

### AURKB knockdown induced cell cycle arrest in CRC cells

**Figure 2:**
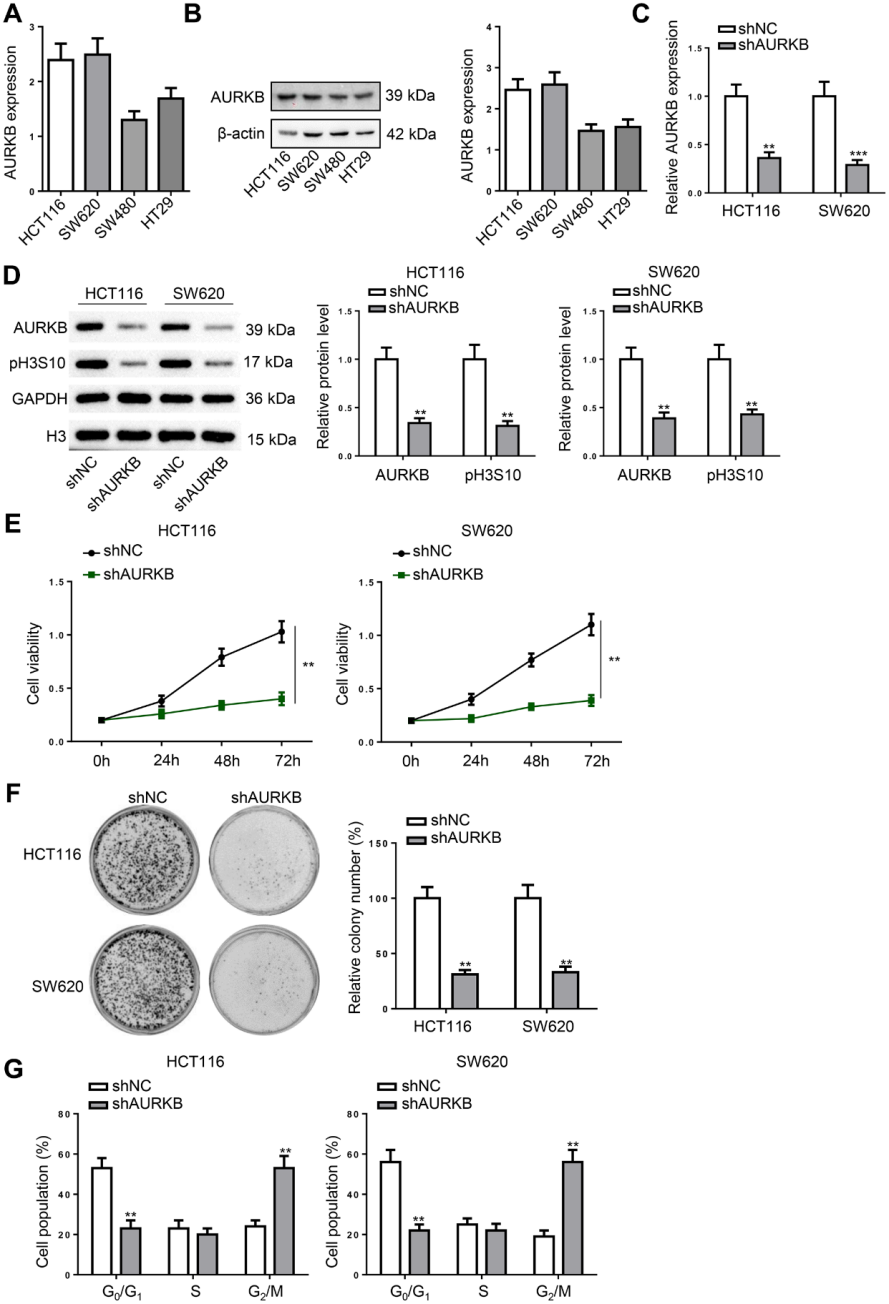
**AURKB knockdown induced cell cycle arrest in CRC cells.** (**A**, **B**) The mRNA and protein expression of AURKB was detected in CRC cell lines (HCT116, SW620, SW480, and HT29) using RT-qPCR and western blot. (**C**, **D**) HCT116 and SW620 cells were transfected with shNC or shAURKB to detect the mRNA expression of AURKB using RT-qPCR (**C**) and the protein level of AURKB and pH3S10 using western blot (**D**). (**E**–**G**) AURKB-silenced HCT116 and SW620 cells were subjected to cell proliferation assessment (**E**), colony formation assay (**F**), and cell cycle analysis (**G**). **p < 0.01, ***p < 0.001.

Subsequently, AURKB was down-regulated in CRC cell lines (HCT116 and SW620) which showed higher AURKB expression (Figure 2A, 2B). Following the treatment, AURKB expression and the phosphorylation of histone H3 at Ser10 (pH3S10) was remarkably reduced (Figure 2C, 2D). It was found that the proliferation of CRC cells was markedly retarded by silencing AURKB (Figure 2E). In addition, AURKB depletion significantly attenuated the colony formation ability of CRC cells (Figure 2F). In cell cycle progression, AURKB knockdown induced G2/M phase arrest (Figure 2G). Altogether, AURKB knockdown inhibits CRC cell proliferation, leading to cell cycle arrest in G2/M phase.

### AURKB targeted CCNE1 in CRC cells

**Figure 3:**
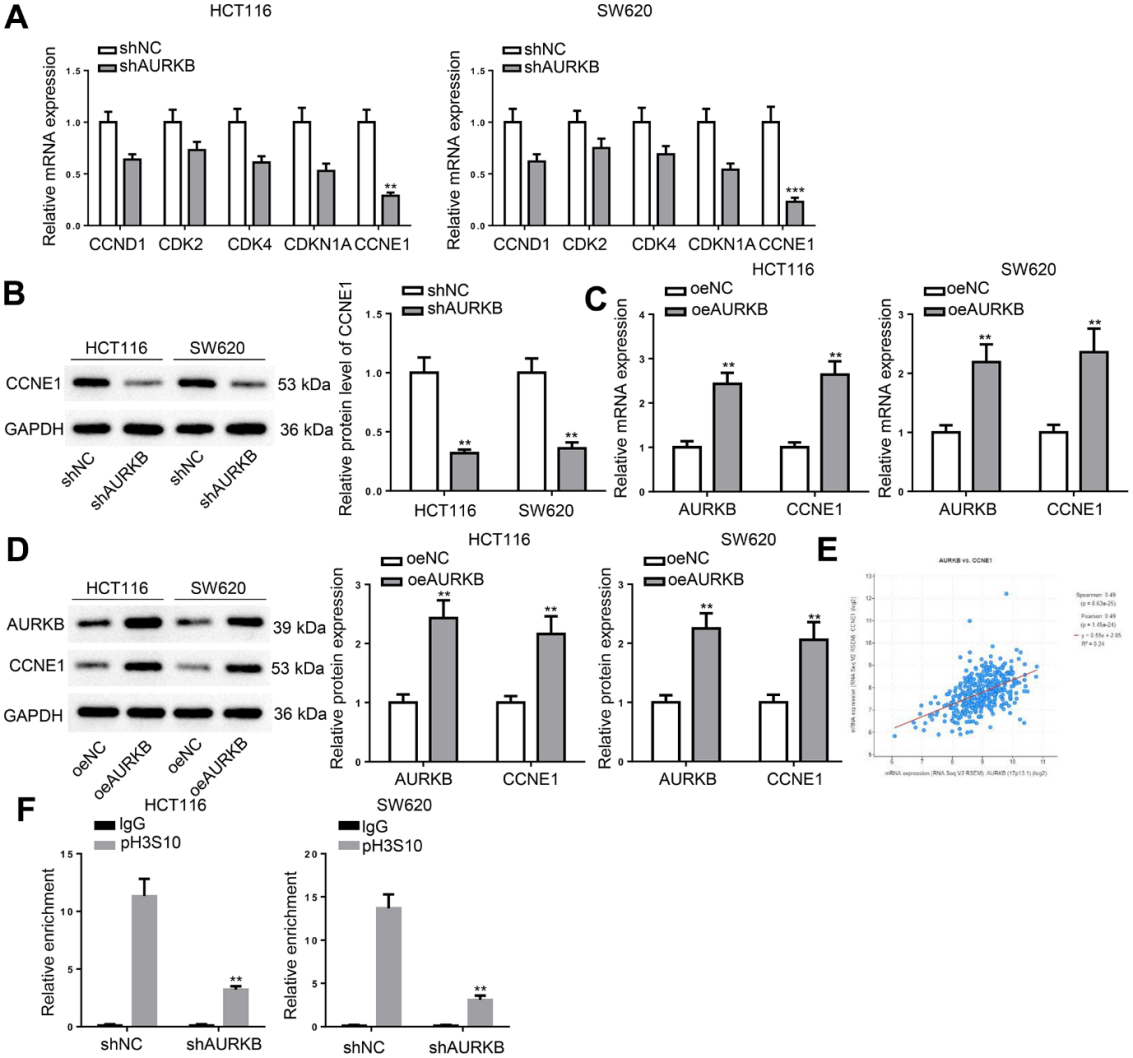
**AURKB targeted CCNE1 in CRC cells.** (**A**) RT-qPCR detected the expression of CCND1, CDK2, CDK4, CDKN1A, and CCNE1 in shNC- or shAURKB-transfected HCT116 and SW620 cells. (**B**) Western blot assessed CCNE1 protein level in shNC- or shAURKB-transfected HCT116 and SW620 cells. (**C**, **D**) The mRNA and protein expression of AURKB and CCNE1 in oeNC- or oeAURKB-transfected HCT116 and SW620 cells. (**E**) The correlation between AURKB and CCNE1 expression in CRC tissues was obtained from TCGA database. (**F**) ChIP assay evaluated the effect of AURKB knockdown on the relative enrichment of pH3S10 in the promoter region of CCNE1. **p < 0.01, ***p < 0.001.

To explore the molecular mechanism underlying the regulatory function of AURKB in the cell cycle of CRC cells, we examined the expression levels of key cell cycle regulatory molecules, including CCND1, CDK2, CDK4, CDKN1A, and CCNE1 in AURKB-silenced CRC cells. RT-qPCR showed that CCNE1 expression was reduced most significantly (Figure 3A), which was consistent with western blot results (Figure 3B). Therefore, we hypothesized that AURKB activate CCNE1 expression in CRC cells. The hypothesis was verified as AURKB overexpression in CRC cells resulted in significant increases in the mRNA and protein expression of CCNE1 (Figure 3C, 3D). Moreover, we noticed that the expression of AURKB and CCNE1 was in a strong positive correlation in CRC tissues (Figure 3E). Considering that AURKB triggers pH3S10 which is generally considered to be associated with the activation of gene expression, a ChIP assay was performed using anti-pH3S10 in AURKB silenced CRC cells. As illustrated in Figure 3F, AURKB knockdown markedly reduced the enrichment of pH3S10 in the promoter region of CCNE1. These results proved that AURKB activated CCNE1 expression in CRC cells.

### AURKB regulated CRC progression via CCNE1 *in vitro*

**Figure 4:**
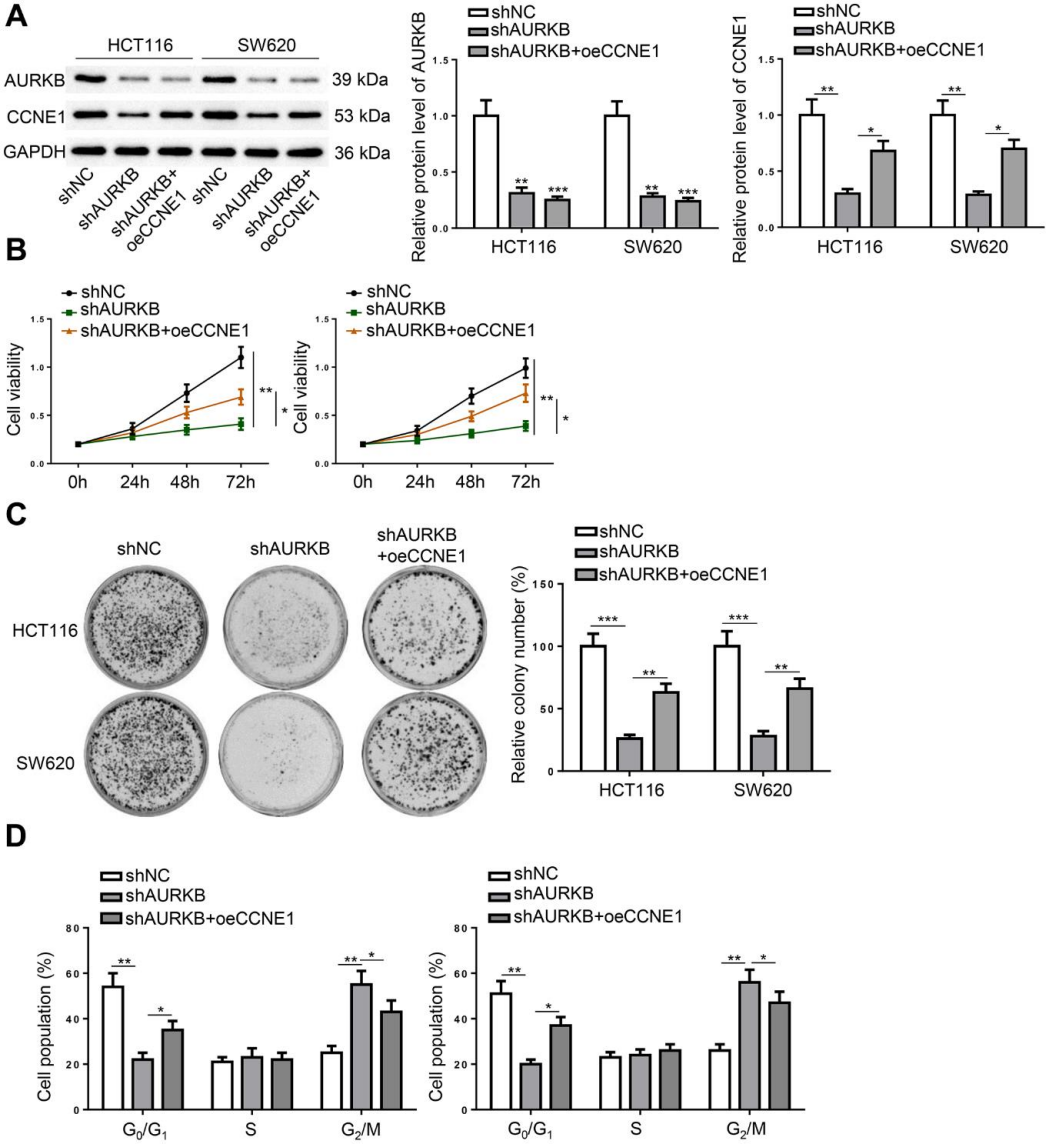
**AURKB regulated CRC progression via CCNE1 *in vitro*.** HCT116 and SW620 cells were divided into three treatment groups, including shNC, shAURKB, and shAURKB + oeCCNE1. (**A**) Western blot detected the expression of AURKB and CCNE1. (**B**–**D**) The proliferation (**B**), colony formation (**C**), and cell cycle (**D**) of the treated cells were evaluated. *p < 0.05, **p < 0.01, ***p < 0.001.

The involvement of CCNE1 in AURKB-mediated CRC progression was confirmed by restoring CCNE1 expression in AURKB-depleted CRC cells. Western blot results indicated that CCNE1 overexpression successfully rescued the deficiency in CCNE1 expression caused by AURKB knockdown, but had no obvious effect on AURKB expression (Figure 4A). Further functional assays showed that forced expression of CCNE1 could partially reversed AURKB knockdown-induced inhibition on cell proliferation, suppression on colony formation, and arrest of cell cycle in G2 phase in CRC cells (Figure 4B–4D). These data suggested that AURKB activated CCNE1 to promote the survival of CRC cells.

### AURKB activated CCNE1 to facilitate CRC tumor growth *in vivo*

**Figure 5:**
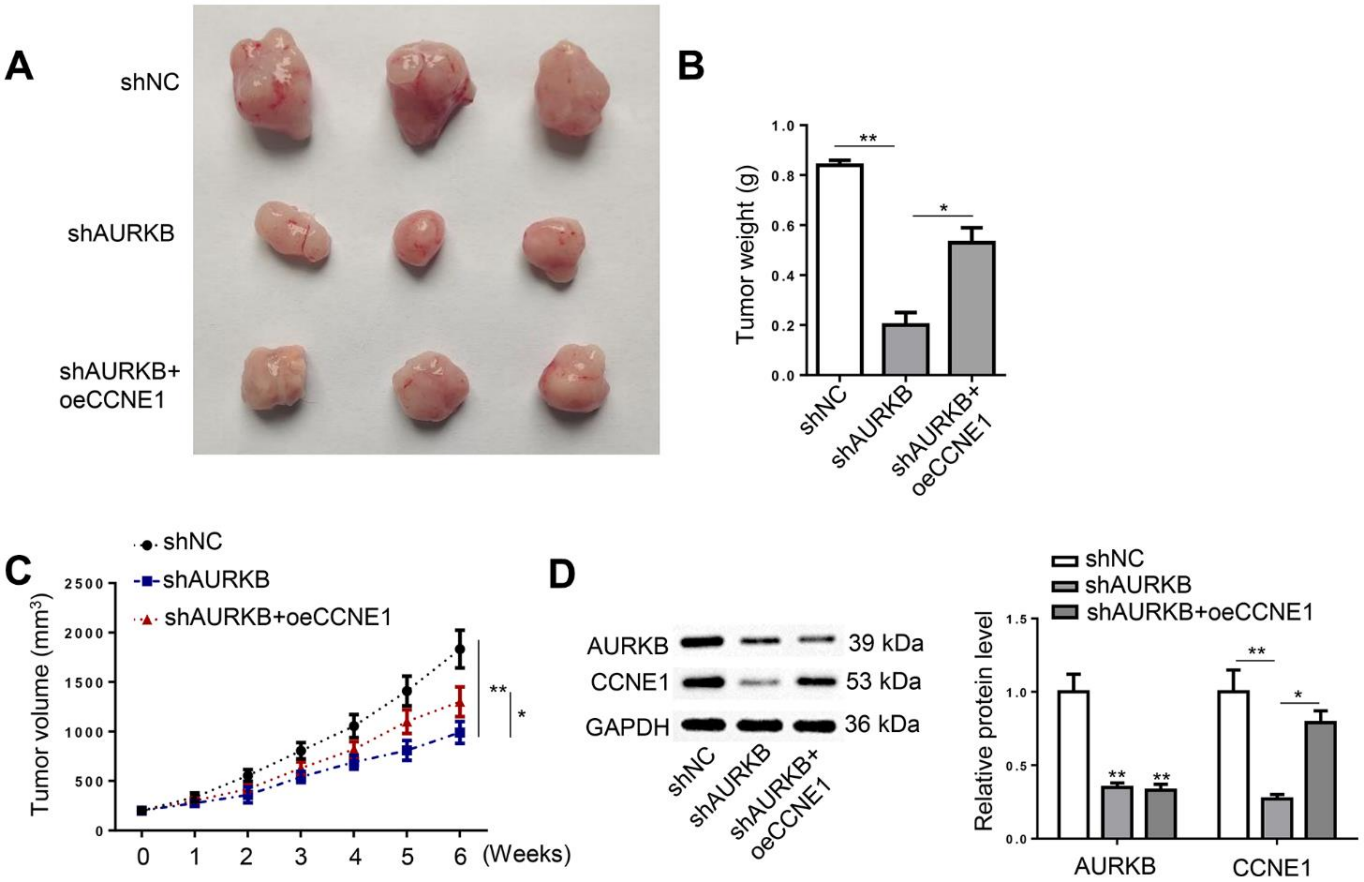
**AURKB activated CCNE1 to facilitate CRC tumor growth *in vivo*.** Xenograft tumors were developed in mice using HCT116 cells treated with shNC, shAURKB, and shAURKB + oeCCNE1. (**A**) Representative images of tumors resected from the experimental mice. (**B**, **C**) Weight and volume of the resected xenograft tumors. (**D**) Western blot detected the expression of AURKB and CCNE1 in the xenograft tumors. *p < 0.05, **p < 0.01.

HCT116 cells depleted with AURKB and supplemented with CCNE1 were applied to establish xenograft tumors in nude mice. It was revealed that the growth of xenograft tumors developed from shAURKB-transfected cells was substantially inhibited compared with the control group; however, the introduction of CCNE1 overexpression readily abrogated the inhibition of AURKB knockdown on tumor growth (Figure 5A–5C). The protein expression of AURKB and CCNE1 in tumor tissues from the three treatment groups were confirmed using western blot (Figure 5D). In sum, AURKB promoted CRC tumor growth through its activation of CCNE1 *in vivo*.

### AURKB inhibition with AZD1152 decreased CCNE1 expression and retarded CRC progression

**Figure 6:**
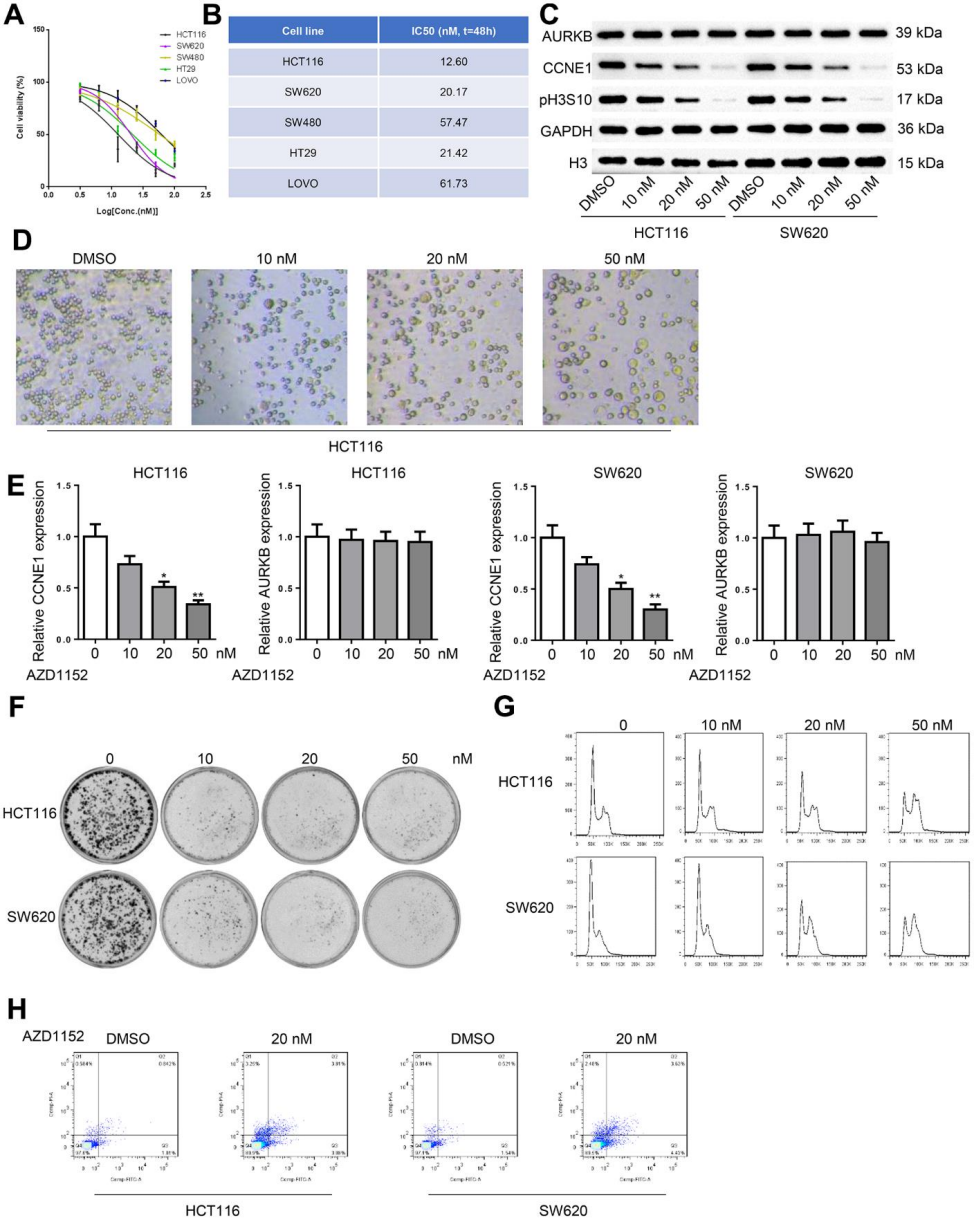
**AURKB inhibition with AZD1152 decreased CCNE1 expression and retarded CRC progression.** (**A**) The viability of CRC cells (HCT116, SW620, SW480, HT29, LOVO) treated with increasing concentrations of AZD1152 was evaluated using CCK-8 assay. (**B**) The IC_50_ value of AZD1152 in CRC cells. (**C**) Western blot detected AURKB and CCNE1 protein level and pH3S10 in HCT116 and SW620 cells treated with 0, 10, 20, 50 nM AZD1152 for 48h. (**D**) The morphology of cells treated with AZD1152 was observed using a microscope. (**E**) The mRNA expression of AURKB and CCNE1 in HCT116 and SW620 cells treated with 0, 10, 20, 50 nM AZD1152 for 48h. (**F**, **G**) HCT116 and SW620 cells treated with 0, 10, 20, 50 nM AZD1152 were subjected to detect colony formation (**F**), cell cycle (**G**). (**H**) The apoptosis of HCT116 and SW620 cells treated with 20 nM was analyzed using flow cytometry. (**H**) *p < 0.05, **p < 0.01.

AZD1152 was proved to be a specific inhibitor for AURKB with anti-cancer effects. The cytotoxic effects of AZD1152 on CRC cells were assessed using CCK-8 assays. As shown in Figure 6A, 6B, the viability of CRC cells was substantially suppressed by AZD1152 in a dose-dependent manner with HCT116 and SW620 cells exhibiting highest sensitivity to AZD1152 treatment. Exposure to AZD1152 inhibited the enzymatic activity of AURKB, causing significant reduction in pH3S10 (Figure 2C), which consequentially resulted in the increases in polyploids (Figure 2D). Expectedly, the expression of CCND1 was decreased consistently, but that of AURKB showed no distinct changes (Figure 2C, 2E). The colony formation capacity of HCT116 and SW620 cells were significantly reduced by AZD1152 (Figure 2F). Flow cytometry analysis revealed a progressive increase of cells in G2/M-phase in CRC cells (Figure 2G). Annexin V/PI double staining showed that 20nM AZD1152 dramatically increased CRC cell apoptosis (Figure 2H). These results indicated that AZD1152 blocked CCNE1 activation and retarded CRC progression.

## DISCUSSION

Aurora kinases (AURKs) are pivotal controllers of the cell cycle, with AURKA and AURKB serving essential roles in mitosis [22], while AURKC plays a notable role in gametogenesis [23]. AURKs possess three distinct domains, with the kinase domain exhibiting a significant level of similarity among all members [23]. The roles of AURKs are clearly delineated through their spatial and temporal expression patterns, as well as the variations in the sequences of their N-terminal regions [24, 25]. Elevated levels of AURKs in tumors have been demonstrated to induce aneuploidy and genomic instability, which in turn promote the development, invasion, and metastasis of tumors [26]. Previous studies have demonstrated that AURKB promoted cancer progression through various mechanism. For example, AURKB accelerates the tumor growth of gastric cancer via activating the expression of cell cycle regulator CCND1 through pH3S10 [27]. AURKB inhibition reduced phospho-histone H3 to attenuate acquired resistance to anti-EGFR therapy in non-small cell lung cancer [28]. AURKB promoted the metastasis of basal-like breast cancer by stabilizing Snail1 to induce epithelial-mesenchymal transition [29]. The current investigation focused on the role of AURKB in CRC.

Through investigating the public databases, the abundant expression of AURKB as well as its positive correlation with Ki-67 expression in CRC tissues were confirmed. *In vitro* experiments indicated that AURKB knockdown suppressed the proliferation and induced G2/M phase cell cycle arrest in HCT116 and SW620 cells. Importantly, we discovered that AURKB triggered pH3S10 to activate CCNE1 expression and the expression of CCNE1 was positively regulated by AURKB.

CCNE1 accumulates during the G1 and S phases, highlighting the significant role of cyclin E1 in facilitating the transition from G1 to S phase in the cell cycle [30]. Elevated levels of cyclin E1 result in an accelerated passage of cells through the G1/S-phase checkpoint, ultimately causing genomic instability [31]. Persistent overexpression of cyclin E in a laboratory setting has been demonstrated to elevate the occurrence of polyploid cells [32, 33]. The irregularities in the way chromosomes separate during cell division raise the likelihood of mutations in other genes responsible for regulating cell survival and growth, thereby driving the affected cells towards a tumorigenic state [34]. Our study proved that CCNE1 overexpression abrogated the suppressive effect of AURKB knockdown on the proliferation and cell cycle progression in CRC cells. Consistently, AURKB deficiency-induced inhibition on the growth of xenograft CRC tumors was reversed by forced expression of CCNE1. It could be summarized that the biological function of AURKB in CRC was exerted through its regulation of CCNE1 expression.

Furthermore, we introduced AZD1152, a specific inhibitor of AURKB [35], to evaluate its effect on AURKB expression and CRC cell survival. AZD1152, which is also referred to as barasertib, exhibits a significantly higher degree of selectivity for AURKB (3000 times more) in comparison to AURKA [36]. We observed that the expression of pH3S10 and CCNE1 was significant suppressed following AZD1152 treatment. In the meanwhile, AZD1152 induced polyploidy, a distinctive characteristic of the phenotypic alterations observed in cells when AURKB is suppressed [37]. In addition, AZD1152 inhibited the proliferation and colony formation while induced G2/M phase cell cycle arrest and cell apoptosis of CRC cell lines, which was consistent with the results obtained by previous studies that exhibited a dose-dependent inhibition of cell proliferation across various cancer types, such as leukemia [38], breast cancer [39], pancreatic and lung cancer [40].

Collectively, it was demonstrated that AURKB triggered pH3S10 to activate CCNE1 expression in CRC cells, thus promoting CRC tumorigenesis. Our result showed the potential oncogenic role of AURKB during the proliferation and cell cycle of CRC cells. Therefore, the current study offers evidence to target AURKB as a therapeutic biomarker in CRC therapy.
